# Serotonin-2C receptor involved serotonin-induced Ca^2+^ mobilisations in neuronal progenitors and neurons in rat suprachiasmatic nucleus

**DOI:** 10.1038/srep04106

**Published:** 2014-02-17

**Authors:** Kouhei Takeuchi, Shahid Mohammad, Tomoya Ozaki, Eri Morioka, Kaori Kawaguchi, Juhyon Kim, Byeongha Jeong, Jin Hee Hong, Kyoung J. Lee, Masayuki Ikeda

**Affiliations:** 1 Graduate School of Innovative Life Science, University of Toyama, 3190 Gofuku, Toyama 930-8555, Japan; 2 Graduate School of Science and Engineering, University of Toyama, 3190 Gofuku, Toyama 930-8555, Japan; 3 Center for Cell-dynamics and Department of Physics, Korea University, Anam-Dong 5-1, Sungbuk-Gu, Seoul 136-713, Korea

## Abstract

The hypothalamic suprachiasmatic nucleus (SCN), the central circadian pacemaker in mammals, undergoes serotonergic regulation, but the underlying mechanisms remain obscure. Here, we generated a subclone of an SCN progenitor cell line expressing Ca^2+^ sensors (SCN2.2YC) and compared its 5-HT receptor signalling with that of rat SCN neurons in brain slices. SCN2.2YC cells expressed 5-HT1A/2A/2B/2C, but not 5A/7, while all six subtypes were expressed in SCN tissues. High K^+^ or 5-HT increased cytosolic Ca^2+^ in SCN2.2YC cells. The 5-HT responses were inhibited by ritanserin and SB-221284, but resistant to WAY-100635 and RS-127445, suggesting predominant involvement of 5-HT2C for Ca^2+^ mobilisations. Consistently, Ca^2+^ imaging and voltage-clamp electrophysiology using rat SCN slices demonstrated post-synaptic 5-HT2C expression. Because 5-HT2C expression was postnatally increased in the SCN and 5-HT-induced Ca^2+^ mobilisations were amplified in differentiated SCN2.2YC cells and developed SCN neurons, we suggest that this signalling development occurs in accordance with central clock maturations.

The suprachiasmatic nucleus (SCN) of the hypothalamus functions as the circadian pacemaker in mammals^1^,^2^. The SCN pacemaker is maternally coupled in the foetus until birth^3^, and develops action potential firing rhythms and entrainability to environmental cues during early postnatal life^4^,^5^,^6^. However, the neuronal mechanisms underlying the development of the circadian clock are not well understood.

In adults, circadian rhythms in SCN neurons are entrained to the environmental light/dark cycle via the glutamatergic retinohypothalamic tract (RHT)^7^. In parallel with the postnatal development of the RHT, the number of astrocytes is increased and the number of neurons is decreased in the SCN^8^,^9^,^10^, suggesting dynamic reorganisation of the SCN circuits or contents in relation to RHT formation. In addition, γ-amino-butyric acid (GABA)-A receptors mediate excitatory synaptic signal transduction in neonatal brains^11^, but are switched to reversible (i.e., excitatory and inhibitory) functions in SCN neurons during postnatal development^12^. The development of GABA-A receptor signalling and intracellular chloride homeostasis may also amplify the circadian action potential firing rhythms in these neurons^13^.

In addition to the above neuronal regulations, the SCN receives dense serotonergic innervations from the midbrain raphe nucleus^14^. The numbers of serotonin (5-HT)-containing axons are greatly increased in the SCN during postnatal life^15^. In adults, 5-HT has been shown to modulate the effects of light by inhibiting glutamatergic RHT synapses in the SCN^14^. However, c-Fos expression in the SCN induced by subcutaneous injection of a 5-HT2A/2C agonist (2,5-dimethoxy-4-iodoamphetamine; DOI) was increased in a slightly different time frame to RHT development in rats^16^, suggesting that differential developmental mechanisms may underlie these systems. In the mature SCN, significant diversity of 5-HT receptor subtypes has been reported for both pre- and post-synaptic sites^17^,^18^,^19^,^20^,^21^,^22^,^23^,^24^,^25^,^26^,^27^,^28^,^29^. However, none of the developmental processes of these 5-HT receptor subtypes have been determined in the SCN to our knowledge.

SCN2.2 cells are immortalised rat SCN progenitor cells created by infection with a retroviral vector encoding the adenovirus 12S E1A gene at embryonic day 18^30^. SCN2.2 cells display (i) extended growth potential without evidence of transformed or tumorigenic properties, (ii) expression of E1A protein within all cell nuclei and (iii) heterogeneous cell types in various stages of differentiation. A large proportion of SCN2.2 cells are characterised by glial cell-like morphologies, but show limited expression of corresponding cell type-specific antigens. Rather, it has been shown that a subpopulation of SCN2.2 cells exhibit neuronal characteristics. Because transplantation of SCN2.2 cells into SCN-lesioned rats recovered their behavioural rhythms^31^ and indeed these cells contain diverse clock genes^32^, it has been proposed that SCN2.2 cells potentially function as substitutive circadian pacemakers, although the cellular component essential for their functions remains unclear. Thus, subcloning of SCN2.2 cells could provide useful tools for studying the development of the SCN and the manifestation of their distinct roles in mammalian circadian timekeeping.

We have developed a method for transfecting yellow cameleon (YC) genes into cultured SCN neurons, thereby enabling monitoring of the circadian cytosolic Ca^2+^ waves in these neurons^33^. In the present study, we generated subclones of SCN2.2 cells expressing YC3.6 and monitored their cytosolic Ca^2+^. Since rhythmic expression of voltage-gated Ca^2+^ channels is a proposed physiological output from SCN2.2 cells^34^, we retrieved a clone using a high-potassium (high K^+^)-induced Ca^2+^ increase as a marker. Here, we report the characteristics of one subclone (SCN2.2YC) with special interest in its 5-HT receptor expressions and functions. The predominant 5-HT receptor subtypes linked to intracellular Ca^2+^ signalling were comparatively examined in SCN2.2YC cells and rat SCN neurons.

## Results

### Profiles of 5-HT receptor expressions in rat SCN punch-outs, SCN astrocytes and SCN2.2 cells

**Figure 1:**
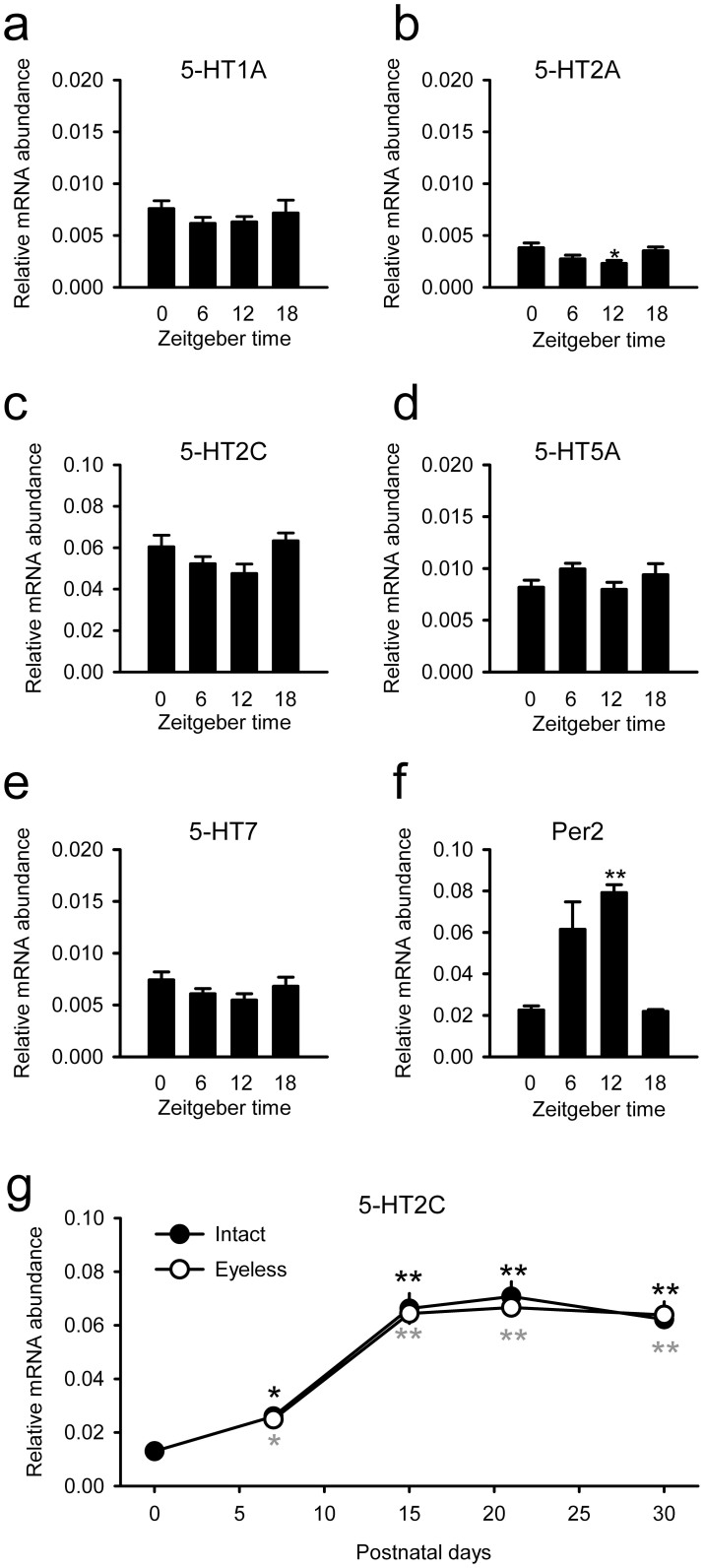
5-HT receptor gene expression profiles in rat SCN punch-outs. (a–e) Day–night differences in the expression levels of 5-HT receptor 1A (a), 2A (b), 2C (c), 5A (d) and 7 (e) subtypes in adult rat SCN punch-outs evaluated by real-time RT-PCR. A housekeeping gene (GAPDH) was evaluated to estimate the relative expression levels. (f) The rhythmic expression of *Per2* within the same sample ensures that the SCN grafts contain the circadian clock. Except for the profile of 5-HT2A receptor expression, the 5-HT receptor expressions are not time-dependent. *n* = 4–5 for each sampling time. *P < 0.05, **P < 0.01 by one-way ANOVA. (g) Developmental differences in the expression levels of 5-HT receptor subtype 2C were analysed in SCN punch-outs of rats that underwent neonatal enucleation surgery (white circles) or a sham operation (black circles). *n* = 4–7 rats per group. *P < 0.05, **P < 0.01 (black asterisks for intact rats and grey asterisks for eyeless rats) versus the day 0 levels by one-way ANOVA followed by Duncan's multiple range test.

**Figure 2:**
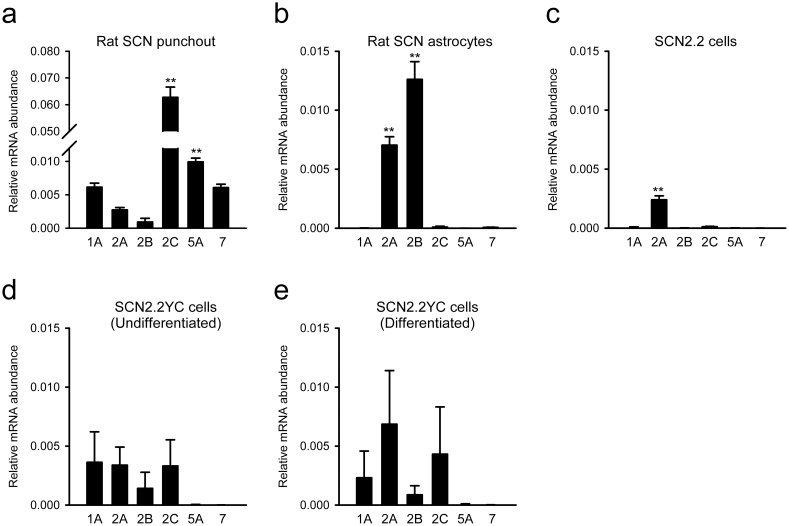
Comparisons of 5-HT receptor gene expression profiles in rat SCN punch-outs, cultured SCN astrocytes and SCN2.2 cells. (a) Averaged expression levels for each 5-HT receptor subtype from the data shown in Fig. 1a–e. (b) Gene expression profiles in primary cultures of astrocytes dissociated from neonatal rat SCN grafts. (c) Gene expression profiles in original (i.e., heterogeneous) SCN2.2 cells under standard culture conditions, demonstrating that 5-HT2A receptors are dominantly expressed in these cells. (d) Subcloned SCN2.2 cells (SCN2.2YC) show higher expression levels of 5-HT1A, 2B and 2C receptors, and are closer to the patterns of SCN punch-outs. (e) Culture of SCN2.2YC cells in 5F differentiation medium elevates the transcriptional levels of these 5-HT receptors, but the differences are not statistically significant. *n* = 4–5 for each sampling time. **P < 0.01 versus the minimal value in the group by Duncan's multiple range tests following one-way ANOVA.

The expressions of various 5-HT receptor subtypes were analysed in punch-outs of the SCN prepared at four different times of the day. The relative expression levels of most of the 5-HT receptor subtypes showed steady transcriptional levels (Fig. 1). The only exception was 5-HT2A receptors, whose transcriptional activities were significantly lower at the time of dark onset (ZT12; Fig. 1b) and showed anti-phased expression rhythms against the clock gene *Per2* transcription rhythms (Fig. 1f). It should be emphasised that there were large variations in the expression levels of the individual 5-HT receptor subtypes. Of the receptor subtypes examined, 5-HT2C showed significantly higher (6–68 times) expression levels than the other subtypes (F_5,24_ = 170; *P* < 0.01 by one-way ANOVA; Fig. 2a). In addition, the 5-HT2C receptor expression levels increased during postnatal development; the signal intensity was five times higher in tissues at postnatal day 15 than in tissues of neonates (F_4,16_ = 36; *P* < 0.01 by one-way ANOVA; Fig. 1g). Since RHT projections are known to be increased during a similar postnatal period, we analysed the possible interaction with retinal inputs to the SCN. However, neonatal enucleation surgery did not modulate the transcriptional levels of 5-HT2C in the SCN (Fig. 1g), indicating that the developmental increase in 5-HT2C receptors occurs regardless of the presence or absence of the RHT.

**Figure 3:**
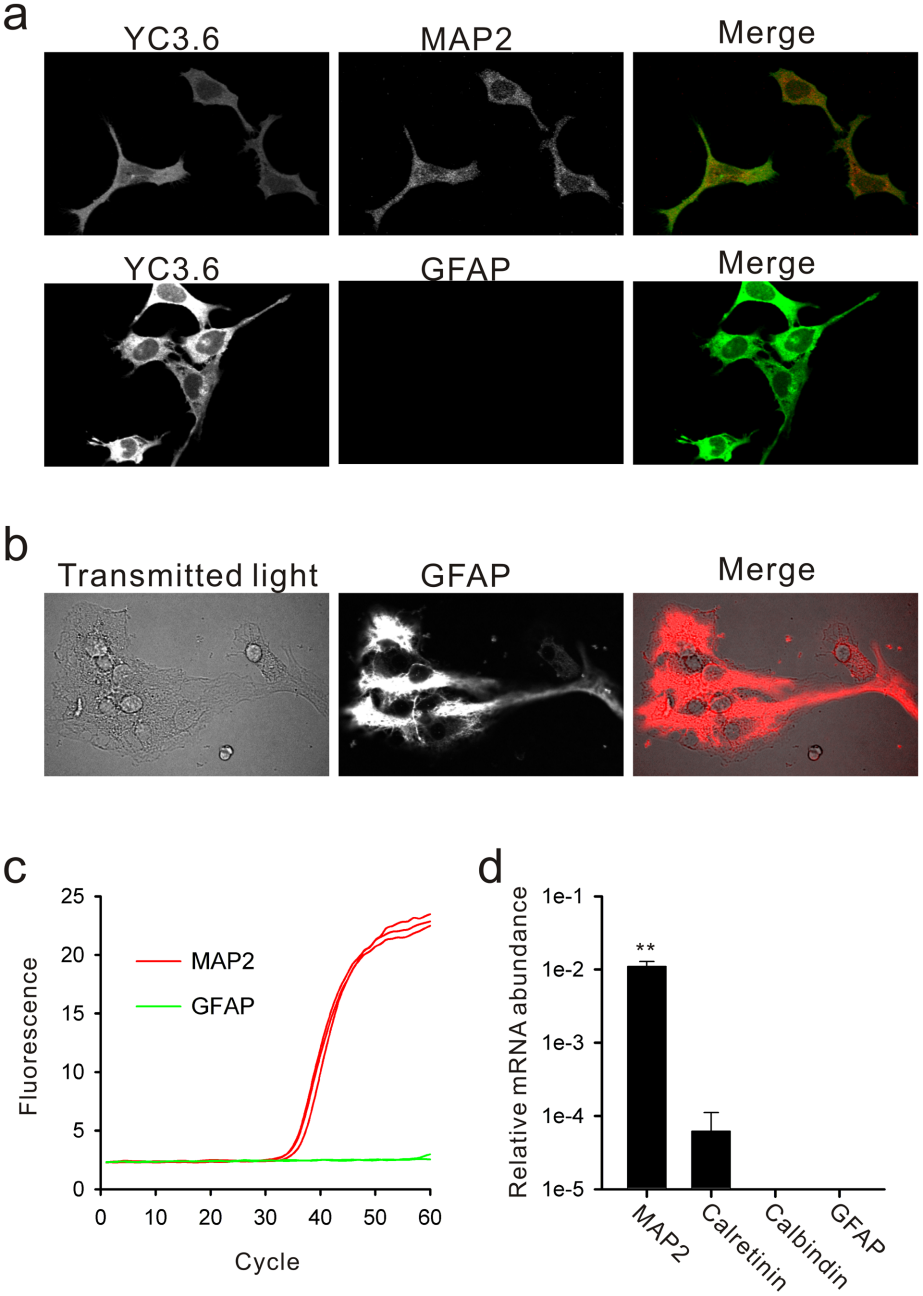
Presence of a neuronal cell marker, but lack of a glial cell marker, in SCN2.2YC cells. (a) A subclone of an SCN progenitor cell line expressing YC3.6 Ca^2+^ sensors (SCN2.2YC) was characterised by immunofluorescence staining for MAP2 (neuronal marker) and GFAP (glial marker). The results show that undifferentiated SCN2.2YC cells express MAP2, but not GFAP. (b) The same GFAP staining for primary cultures of astrocytes reveals strong fibrous staining. (c) Real-time RT-PCR assays further demonstrate consistent transcription levels for MAP2 and GFAP (real-time PCR processes shown in red and green, respectively) in undifferentiated SCN2.2YC cells. These analyses are based on entire cell extracts from culture flasks. (d) In addition, the transcriptional levels of two neuronal Ca^2+^-binding proteins, calretinin and calbindin-D28k, were analysed, and found to be low or none. The relative mRNA levels were quantified by the level of GAPDH mRNA. **P < 0.01 by one-way ANOVA followed by Duncan's multiple range test.

The expression patterns of diverse 5-HT receptor subtypes were also examined in primary cultures of rat SCN astrocytes. The SCN astrocytes displayed expression of the 2A and 2B subtypes, with higher levels of the 2B subtype (Fig. 2b). This expression pattern did not fit with either the total SCN tissues or SCN2.2 cells (Fig. 2c). The original (i.e., heterogeneous) SCN2.2 cells displayed relatively low expression levels of 5-HT receptors, with only the 2A subtype showing equivalent levels to the SCN punch-outs (Fig. 2c). However, the SCN2.2YC subclone showed higher expression levels of the 1A subtype (*P* < 0.05 by two-tailed Student's *t*-test) and 2C subtype (*P* < 0.05 by two-tailed Student's *t*-test) compared with the original SCN2.2 cells (Fig. 2d). Differentiation of SCN2.2YC cells with five factors-supplemented (5F) medium increased the transcriptional levels of these 5-HT receptor subtypes, but the differences were not statistically significant (Fig. 2e). The characteristics of the SCN2.2YC clone were more like those of neuronal progenitors than glial progenitors, because SCN2.2YC cells expressed a neuronal cell marker, microtubule-associated protein 2 (MAP2), but not a glial cell marker, glial fibrillary acidic protein (GFAP) (Fig. 3).

### 5-HT-induced responses in rat SCN neurons

**Figure 4:**
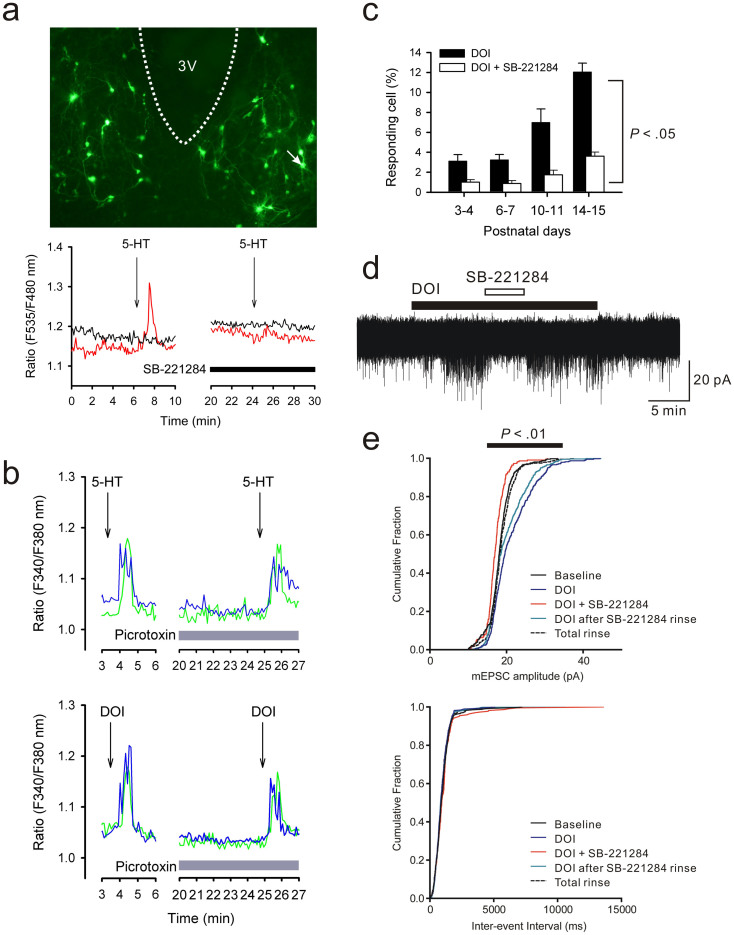
Post-synaptic 5-HT responses in the rat SCN. (a) *Upper*, Representative fluorescence image of a rat SCN slice culture expressing NSE promoter-driven YC3.6. 3 V, third ventricle. *Lower*, 5-HT evokes a Ca^2+^ transient in neurons. The black arrow indicates the timing of a 45-sec pulse of 5-HT (3 μM). Each coloured trace denotes the cytosolic Ca^2+^ level in an individual neuron. A neuron that displayed the 5-HT-induced Ca^2+^ increase (red trace) was located at the ventrolateral SCN (marked by the white arrow on the upper image). The 5-HT-induced Ca^2+^ increase was inhibited by the 5-HT2B/2C antagonist SB-221284 (300 nM). (b) A similar experiment with *fura-2*-stained acute SCN slices prepared from rats. In this experiment, a second 5-HT stimulation was conducted under perfusion of a GABA-A receptor antagonist, picrotoxin (50 μM), but the effects are limited. Similar Ca^2+^ transients are also produced by a 5-HT2A/2C agonist, DOI (1 μM). (c) The DOI-induced Ca^2+^ response is increased during postnatal development and inhibited by 300 nM SB-221284 (5-HT 2B/2C receptor antagonist). A total of 8–12 slices were used for each trial. *P* < 0.05 by two-way ANOVA. The recordings in (b) and (c) were examined under perfusion of TTX (0.5 μM) throughout the experimental period. (d) Voltage-clamp recording of acute SCN slices, showing enhancement of miniature EPSCs by DOI (1 μM) and antagonisation of the DOI action by SB-221284 (500 nM). (e) EPSC amplitudes rather than the frequencies are increased by DOI (*P* < 0.01 by two-way ANOVA). These results indicate post-synaptic 5-HT2C receptor expressions in the rat SCN.

The neuronal responses against 5-HT or DOI were examined using rat SCN slices. First, SCN slice cultures, which were prepared from 3-day-old mouse pups and expressed neuron-specific enolase promoter-driven (NSEp)-YC3.6, were stimulated with 5-HT (3 μM, 45 sec) under perfusion of 0.5 μM tetrodotoxin (TTX). Under these conditions, 5% of SCN neurons (4 of 82 neurons/4 slices) displayed Ca^2+^ transients (Fig. 4a). An antagonist for 5-HT2B/2C, SB-221284 (300 nM), completely blocked the 5-HT-induced Ca^2+^ increase (Fig. 4a). Similarly, in *fura-2*-loaded SCN slices, which were acutely isolated from rats (postnatal days 10–12), 5-HT (3 μM, 45 sec) evoked Ca^2+^ transients in 7% of cells under perfusion of TTX (15 of 211 cells/7 slices) and the responses were not inhibited by a GABA-A receptor antagonist (picrotoxin; 50 μM) (Fig. 3b). In addition, DOI (1 μM, 45 sec) evoked Ca^2+^ transients in 7% of cells under both perfusion of TTX (14 of 199 cells/6 slices) and TTX with picrotoxin (15 of 215 cells/6 slices) (Fig. 3b). The occurrence of DOI-induced Ca^2+^ mobilisations in acute SCN slices was dependent on developmental timing, since it was only observed in about 3% of the total cells during the first week after birth, but reached 12% of the population (24 of 198 cells/7 slices) at 2 weeks of age (Fig. 4c). The DOI responses at all stages were significantly (−68% to −75%) inhibited by SB-221284 (Fig. 4c). Taken together, these results demonstrate the involvement of 5-HT2C receptors in the Ca^2+^ mobilisations in SCN cells dependently on postnatal development, but independently of synaptic interactions within the circuits.

The functions of 5-HT receptors at the glutamatergic synapses of SCN neurons were further analysed by monitoring miniature excitatory postsynaptic currents (EPSCs) using voltage-clamp electrophysiology in acute SCN slices of postnatal days 12–16. In a subpopulation of SCN neurons that displayed spontaneous miniature EPSCs (6 of 28 neurons; average amplitude = 18.2 ± 0.2 pA), DOI (1 μM) magnified the miniature EPSC amplitude in half of the neurons (average amplitude = 21.3 ± 0.3 pA; *P* < 0.01 by a two-tailed Student's *t*-test; n = 3; Fig. 4d), but had no effects on the frequency (Fig. 4e). In addition, co-perfusion of SB-221284 (500 nM) antagonised the DOI actions on the EPSC amplitudes in these neurons. These results suggest the presence of 5-HT2C receptors in SCN neurons at post-synaptic sites of glutamate synapses.

### 5-HT-induced Ca^2+^ transients in SCN2.2YC cells

**Figure 5:**
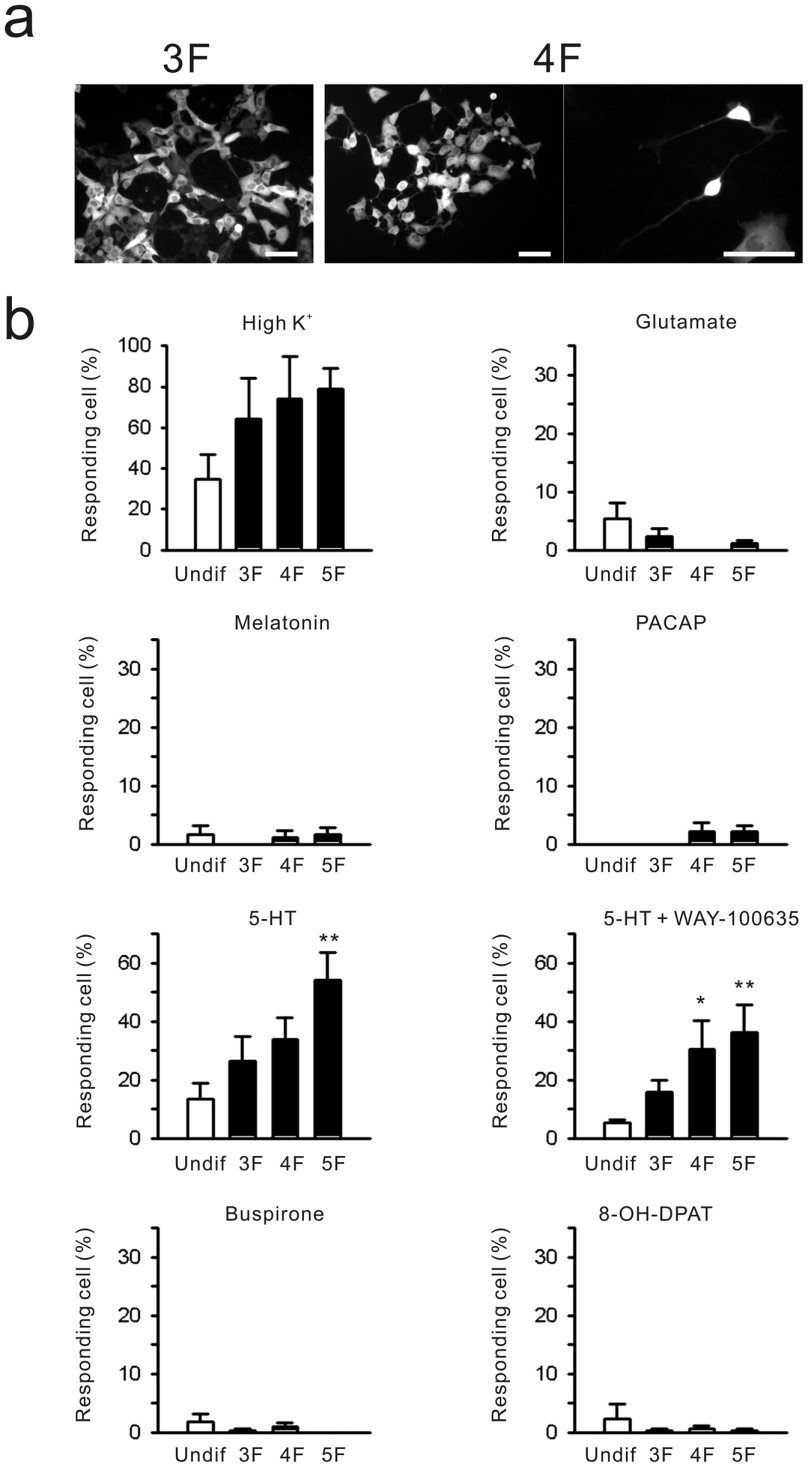
Cytosolic Ca^2+^ responses in SCN2.2YC cells under different culture conditions. (a) *Left*, Fluorescence (F535 nm) image of SCN2.2YC cells cultured with insulin, transferrin and sodium selenite (3F), which are indistinguishable from control culture conditions. *Middle*, Image of SCN2.2YC cells cultured in medium supplemented with cholera toxin (4F). *Right*, Enlarged image. Note that polarised neuronal shapes are produced after addition of cholera toxin. (b) Cell population ratios showing increases in intracellular Ca^2+^ concentrations following high potassium (High K^+^) or various receptor stimulations compared among different cell culture conditions. High K^+^ induces intracellular Ca^2+^ flux in 35% of undifferentiated (Undif) SCN2.2YC cells. The response is elevated by more than two-fold following cell differentiation by the 5F medium (4F medium plus PMA). However, SCN2.2YC cells show no or little responsiveness to glutamate (1 mM), PACAP (100 nM) and melatonin (1 μM), regardless of the culture conditions. 5-HT (3 μM) triggers Ca^2+^ transients, and this response is amplified by differentiation media. The dominant 5-HT response is insensitive to WAY-100635 (100 nM), an antagonist for 5-HT1A/7 receptors. Consistently, a 5-HT1A agonist (buspirone; 10 μM) and 5-HT 1A/7 agonist (8-OH-DPAT; 1 μM) have limited actions in SCN2.2YC cells. *n* = 6–12 per group. *P < 0.05, **P < 0.01 by one-way ANOVA.

**Figure 6:**
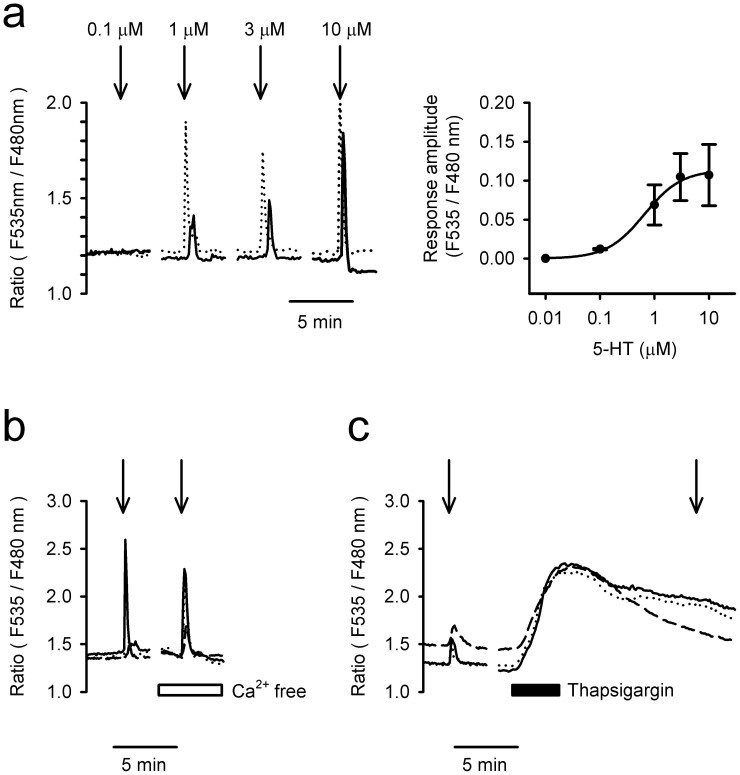
Characteristics of 5-HT-induced cytosolic Ca^2+^ mobilisations in 5F-differentiated SCN2.2YC cells. (a) *Left*, Representative 5-HT-induced Ca^2+^ transients in two SCN2.2YC cells. A dose-dependent increase in intracellular Ca^2+^ by 5-HT is observed in differentiated SCN2.2YC cells. *Right*, Dose-response curve for the 5-HT-induced increase in cytosolic Ca^2+^ in SCN2.2YC cells. (b) The 5-HT (1 μM, 45 sec)-induced Ca^2+^ response is resistant to extracellular Ca^2+^-free buffer. (c) Conversely, the 5-HT response is abolished after reduction of internal stores by thapsigargin (1 μM). All of the above responses were repeatedly observed in at least 50 cells in three independent trials.

**Figure 7:**
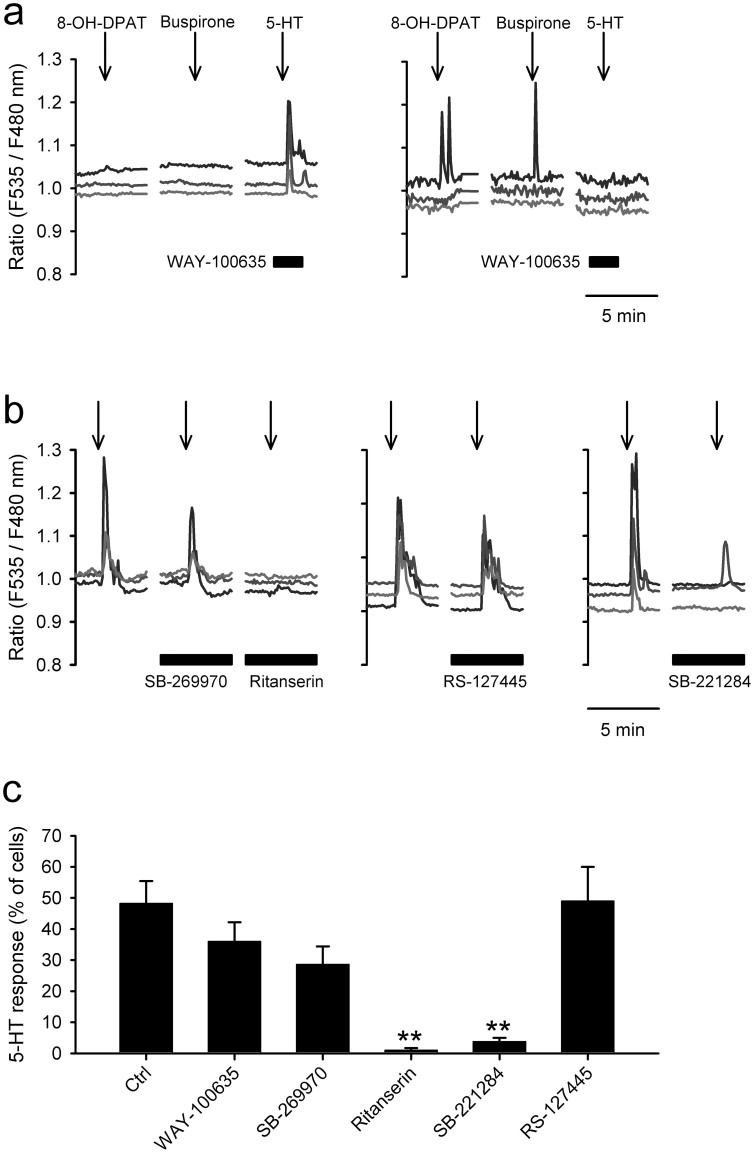
Pharmacological blockade of 5-HT-induced Ca^2+^ mobilisations in 5F-differentiated SCN2.2YC cells. (a) SCN2.2YC cells were stimulated for 45 sec with a 5-HT1A/7 agonist (8-OH-DPAT; 1 μM), 5-HT1A agonist (buspirone; 10 μM) and 5-HT (1 μM) with a 5-HT 1A/7 antagonist (WAY-100635; 100 nM). *Left*, The majority (36%) of SCN2.2YC cells respond to 5-HT under perfusion of WAY-100635, showing no responses to 8-OH-DPAT and buspirone. *Right*, A few cells (2%) show reversed responsiveness against the stimulants. (b) Cells were repeatedly (2–3 times/dish) stimulated with 5-HT (1 μM, 45 sec) at 20-min intervals. The second or third stimulations were examined under perfusion of 5-HT antagonists: 5-HT 7 receptor antagonist (SB-269970; 30 nM); 5-HT 2 receptor antagonist (ritanserin; 30 nM); 5-HT 2B receptor antagonist (RS-127445; 100 nM); or 5-HT 2B/2C receptor antagonist (SB-221284; 100 nM). The 5-HT induced Ca^2+^ transients are significantly reduced by ritanserin or SB-221284. (c) The means were calculated from 13–20 independent trials. **P < 0.01 versus the control response by Duncan's multiple range tests following one-way ANOVA.

During the course of SCN2.2 cell subcloning, we selected a clone by monitoring a 60-mM high-K^+^-induced Ca^2+^ increase as an index of neural responses. The resultant monoclonal SCN2.2YC cells displayed the high-K^+^-induced Ca^2+^ increase in 35% of cells (97 of 276 cells/12 dishes; Fig. 5). The occurrence of the high-K^+^-induced Ca^2+^ response tended to be higher in differentiated SCN2.2YC cells, although there were large variations in each trial (F_3,21_ = 2.68; N.S. by one-way ANOVA). Stimulation of SCN2.2YC cells with glutamate (1 mM), melatonin (100 nM) or pituitary adenylate cyclase-activating polypeptide (PACAP; 1 μM) failed to increase cytosolic Ca^2+^ under nearly all cell culture conditions examined. Conversely, 5-HT (3 μM) induced Ca^2+^ transients in 13% of cells in undifferentiated conditions (17 of 132 cells/5 dishes). In addition, the occurrence of 5-HT-induced Ca^2+^ responses was 4.4 times higher in 5F-differentiated conditions (F_3,31_ = 6.1; *P* < 0.01 by one-way ANOVA). The 5-HT-induced Ca^2+^ responses in SCN2.2YC cells were dose-dependent, with an EC_50_ of 660 nM (Fig. 6a). The 5-HT-induced Ca^2+^ responses were resistant to perfusion of Ca^2+^-free buffer (Fig. 6b), but abolished by depletion of internal Ca^2+^ stores by thapsigargin (Fig. 6c), demonstrating Ca^2+^ release from internal Ca^2+^ stores via 5-HT receptor stimulations. Most of the 5-HT-induced Ca^2+^ responses in SCN2.2YC cells were not blocked by a 5-HT1A/7 antagonist, WAY-100635 (Figs. 5 and 7a). Consistently, a 5-HT1A agonist, buspirone (4 of 144 cells/5 dishes), or 5-HT1A/7 agonist, 8-OH-DPAT (4 of 135 cells/5 dishes), mimicked the effects of 5-HT in less than 3% of cells (Figs. 5 and 7a). Furthermore, the occurrence of the 5-HT-induced Ca^2+^ responses was significantly blocked by a general 5-HT2 receptor antagonist, ritanserin, or SB-221284 (F_5,86_ = 9.1; *P* < 0.01 by one-way ANOVA), but not by an antagonist for 5-HT2B, RS-127445 (Fig. 7b,c). These series of pharmacological examinations indicate that the 5-HT-induced Ca^2+^ increases are predominantly mediated by 5-HT2C receptors in SCN2.2YC cells.

### Circadian oscillatory profiles of SCN2.2YC cells

**Figure 8:**
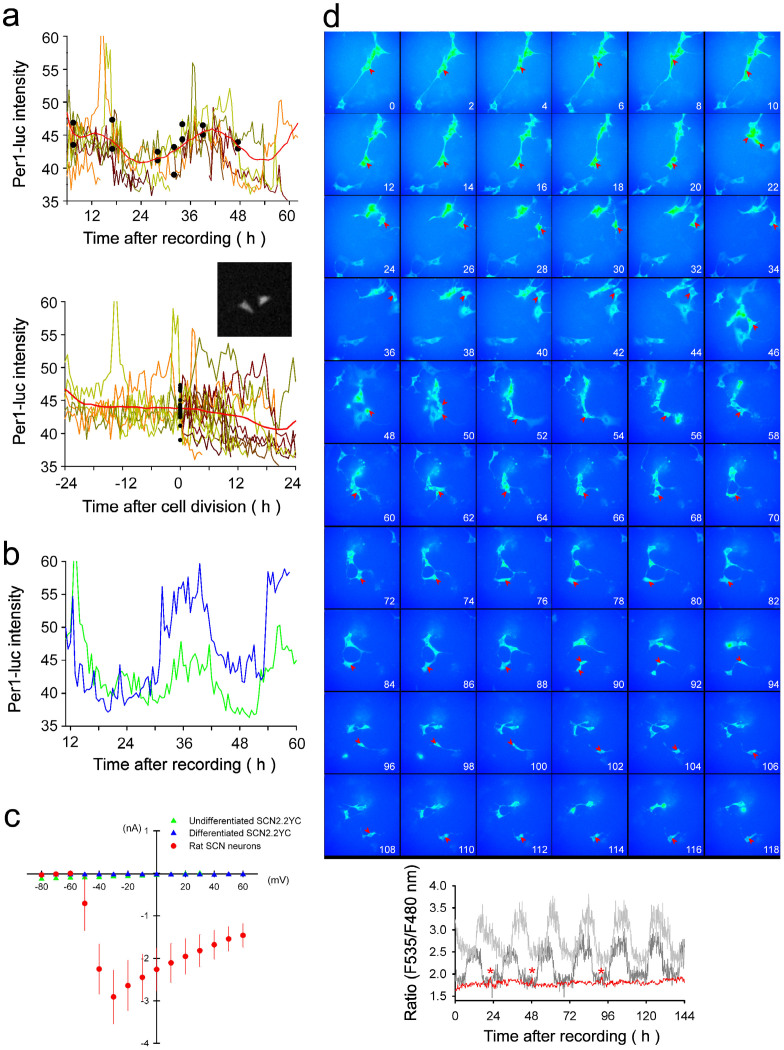
Presence of molecular clock oscillations, but lack of apparent physiological outputs, in SCN2.2YC cells. (a) *Per1* transcriptional rhythms in SCN2.2YC cells were analysed by *Per1*-luciferase chemiluminescence imaging. *Upper*, The circadian rhythms in individual undifferentiated SCN2.2YC cells (individual earth colour traces) are sustained for several days in a culture dish. The averaged intensity is plotted in red. Each cell oscillation is synchronised, presumably through the medium exchange immediately before starting the video microscopy. Black dots denote times of cell divisions. *Lower*, The same data re-plotted as a function of cell division time. The averaged intensity (red) fails to display circadian oscillations. This indicated circadian phase of *Per1* transcriptional rhythms does not depend on the time of cell division. The representative image demonstrates the chemiluminescence of daughter cells immediately after cell division. (b) Representative *Per1* transcriptional rhythms in undifferentiated (green) and 5F-differentiated SCN2.2YC cells (blue). Although the transcription levels are slightly higher in the differentiated cells, the rhythmic profiles are similar between the cells. (c) Voltage-dependent Na^+^ currents analysed by whole-cell patch-clamp recordings from rat SCN neurons (red), undifferentiated SCN2.2YC cells (green) and 4F-differentiated SCN2.2YC cells (blue). A total of 4–8 cells were recorded for each group. Note that only the mature neurons display voltage-dependent Na^+^ currents. (d) Long-term YC3.6 imaging in SCN2.2YC cells. Recordings were conducted in 4F medium. The red arrowheads indicate a cell successfully followed for 6 days. The estimated intracellular Ca^2+^ levels as changes in the fluorescence ratio are plotted in the red trace at the bottom. The red asterisks denote the times of cell divisions. The two grey traces denote the changes in two representative SCN neurons in a rat slice culture. Note that there are no circadian oscillations in the cytosolic Ca^2+^ concentrations in SCN2.2YC cells.

In the present study, we evaluated whether circadian rhythmic components are present in SCN2.2YC cells. First, chemiluminescence imaging of *Per1*-luciferase was used to visualise the presence of *Per1* transcriptional rhythms in undifferentiated SCN2.2YC cells (Fig. 8a). The circadian phase of each cellular rhythm did not depend on the time of cell division (Fig. 8b), consistent with the characteristic clock gene transcriptional rhythms. In addition, the cell differentiation by 5F medium did not modulate the *Per1*-luciferase rhythms, although the basal transcription levels tended to be higher in the medium (Fig. 8b). These results demonstrate the presence of clock gene transcriptional rhythms in both undifferentiated and differentiated SCN2.2YC cells. Conversely, voltage-dependent sodium channels were not expressed or not functional in undifferentiated (*n* = 6) and 4F-differentiated (*n* = 8) SCN2.2YC cells, since the voltage steps failed to evoke typical inward currents (Fig. 8c). These findings demonstrate the lack of action potential firings in SCN2.2YC cells under our cell culture conditions. In addition, long-term ratiometric fluorescence measurements were used to visualise the cytosolic Ca^2+^ levels for 6 days in SCN2.2YC cells (*n* = 8 for undifferentiated cells; *n* = 10 for 4F-differentiated cells), but there were no circadian oscillations in the baseline Ca^2+^ levels (Fig. 8d). These results demonstrate few neuronal output rhythms in SCN2.2YC cells under the cell culture conditions examined.

## Discussion

The SCN is a tightly packed neuronal nucleus composed of approximately 10,000 heterogeneous neurons, and the receptor expression profile in each neuron is generally almost inaccessible. The 5-HT system is not an exception, and diversity of 5-HT receptor subtypes has been reported for both pre- and post-synaptic sites^17^,^18^,^19^,^20^,^21^,^22^,^23^,^24^,^25^,^26^,^27^,^28^,^29^. The involvement of 5-HT2C receptors in circadian clock regulation has been shown at the system level^16^,^17^, but there was no evidence regarding the receptor localisations involved. The present study demonstrated significant expression of 5-HT2C receptors in the rat SCN, which increased during postnatal development but was independent of RHT development. The 5-HT2C receptors were post-synaptically expressed in SCN neurons because the EPSC amplitudes, but not frequencies, were increased following receptor stimulations. In addition, stimulation of 5-HT2C receptors mobilised intracellular Ca^2+^ in SCN cells in rat brain slices with a larger response in juveniles than in neonates. Consistently, a monoclonal rat SCN progenitor cell line, SCN2.2YC, presented Ca^2+^ mobilisations predominantly via 5-HT2C receptors with a larger response in differentiated cells. Taken together, we suggest that the development of 5-HT2C-mediated cellular responses occurs in parallel with the maturation of the central circadian clock. On the other hand, since neither functional voltage-dependent sodium channels nor circadian cytosolic Ca^2+^ waves were present in differentiated SCN2.2YC cells, it is also suggested that the expression of 5-HT2C receptors is not a determinant for the development of physiological rhythms in SCN neurons.

During the course of the Ca^2+^ imaging analyses of SCN2.2YC cells in the present study, we found 5-HT-induced Ca^2+^ transients in these cells. Similar 5-HT-induced Ca^2+^ responses were reported in *fura-2AM*-stained rat SCN slices^35^, although the receptor subtypes involved were not analysed in that study. Therefore, to further analyse the coherency with the rat SCN, the expression profiles of the 5-HT receptor subtypes were examined in detail using real-time RT-PCR. Although 5-HT1B receptors are known to be involved in photic regulation of circadian behavioural rhythms, 5-HT1B receptors have been shown to be localised at pre-synaptic terminals of the RHT or other axon terminals^23^,^36^,^37^. Since the principal aim of this study was to characterise the expression of the 5-HT receptor subtypes in SCN cells, we focused on all other 5-HT receptor subtypes known to be expressed in SCN neurons or to regulate circadian behavioural rhythms. Within the rat SCN punch-outs, 5-HT1A, 2A, 2B, 2C, 5A and 7 subtypes were all expressed, but showed little circadian variations in their expression levels. Notably, we observed significant expression of the 2C subtype among these subtypes. This observation is consistent with the present results showing inhibition of 5-HT (or DOI)-induced Ca^2+^ elevations by SB-221284 in rat SCN slices. In addition, these results are consistent with immunohistochemical analyses of the rat SCN, which identified high levels of 5-HT2C receptors in SCN neurons^19^.

It has also been shown that activation of 5-HT2C receptors acutely induces *Per* gene expression in the rat SCN *in vitro* and *in vivo*^38^,^39^. Conversely, 5-HT-induced phase-shifts of the action potential firing rhythms were observed in SCN slices of 5-HT7 receptor knockout mice, which were reduced by either WAY-100635 or ritanserin^40^. These observations indicate the co-contributions of 1A and 2 receptors to the production of circadian phase-shifts in mice. Furthermore, it has been shown that a 5-HT1A receptor agonist (MKC-242) potentiated, while 8-OH-DPAT inhibited, the photic entrainment of circadian behaviours in hamsters^18^,^41^. Since the effect of MKC-242 on the photic entrainment was blocked by WAY-100635, but not by ritanserin, in the hamster SCN, it was suggested that 5-HT1A receptors, but not 5-HT2 subtypes, are involved in the photic regulation of the hamster circadian clock^18^. Thus, it should be noted that the differences in involvement of each 5-HT receptor subtype or expression at pre- or post-synaptic sites to control the system-wide clock effects may be variable among animal species.

In SCN2.2YC cells, expression of 5-HT5A and 7 receptors was not detected. Because SCN2.2YC cells are monocloned cells, these results may simply arise through subtraction of a characteristic cell population from the heterogeneous SCN cells. However, it was to our surprise that SCN2.2YC cells still showed diversity in their expressions of 5-HT receptor subtypes. These results suggest that the expressions of 5-HT receptor subtypes may not be pre-programmed at the progenitor stages and could be modified during developmental processes or by cell culture conditions. In this regard, the present study also demonstrated gradual increases in 5-HT2C receptor expression in rats during early postnatal development (postnatal days 3–11), regardless of the presence or absence of the RHT. These observations are consistent with a former report showing that c-Fos expression evoked by subcutaneous injection of DOI was increased during postnatal development in a slightly different time frame to RHT development in rats^16^. Since 5-HT-containing axons are greatly increased in the SCN during the corresponding period in early postnatal life^15^, the increases in 5-HT2C receptor expression and maturation of other neuronal functions may arise through interaction with the developmental process of 5-HT-containing axons projecting from the midbrain raphe nucleus.

To address the above hypothesis, we preliminarily examined 5-HT (1 μM) supplementation in the culture medium to observe the changes in the 5-HT receptor expression levels in SCN2.2YC cells, but did not detect any changes in these levels. Therefore, we used a conventional strategy to differentiate these cells. We initially examined differentiation methods with dibutyryl-cAMP or retinoic acid, but failed to differentiate SCN2.2 cells using these reagents. Thus, we used the 4F medium method that successfully differentiated neural progenitor cell lines derived from the cerebellum of a *p53*-deficient mouse^42^. Addition of chorea toxin, an activator of G_s_-mediated signalling, may be critical for the cell differentiation, because the cell divisions were greatly inhibited, while the cell polarisations were greatly enhanced, by chorea toxin. In addition, we added phorbol-12-myristate 13-acetate (PMA), an activator of protein kinase C signalling, into the 4F medium (5F) and observed further enhancements of the 5-HT responses. However, it should be noted that these artificial cell differentiation protocols still failed to increase the 5-HT2C receptor gene expression levels up to the level observed in the adult SCN. Furthermore, these protocols failed to produce voltage-dependent sodium currents, which are essential for the production of action potential firings. Thus, we speculate that other critical factors may be needed for more complete neural differentiation of SCN progenitor cell lines.

To analyse the oscillatory profiles of SCN2.2YC cells in the present study, we examined *Per1*-luciferase and Ca^2+^ imaging. The *Per1*-luciferase imaging in SCN2.2YC cells demonstrated circadian transcriptional rhythms of *Per1* in individual cells and each phase synchronised in the culture dish, but not with the time of cell division. Phase-uncoupling between *Per1* transcriptional rhythms and cell division cycles is consistent with the characteristics of clock gene transcriptional rhythms in NIH3T3 fibroblasts^43^. Notably, our *Per1*-luciferase imaging demonstrated that the circadian amplitude was small and the waveform did not obey a typical sinusoidal wave in each SCN2.2YC cell, unlike the fibroblast models. These observations may not simply be caused by the technical limitations of our imaging system, because the same system demonstrated robust circadian waves in the *Per*-luciferase signals in single cells in *Drosophila* culture models^44^. Thus, we suggest that the clock gene transcriptional rhythms are weaker in SCN2.2YC cells than in fibroblasts or in cells located in the real clock system. The circadian amplitude and phase of the *Per1*-luciferase rhythms in SCN2.2YC cells were not significantly modified by cell differentiation. Thus, although functional 5-HT receptors are present, it seems likely that SCN2.2YC cells may be immature as circadian pacemakers. The majority of cultured SCN neurons display robust circadian cytosolic Ca^2+^ waves^33^,^45^,^46^,^47^, but this did not occur in SCN2.2YC cells. Because Ca^2+^ triggers clock gene transcriptions^48^, circadian cytosolic Ca^2+^ waves could represent feedback signals to the clock gene transcriptional cycles^49^,^50^,^51^. Therefore, the relatively weak *Per1* rhythms observed in SCN2.2YC cells may be explained by the lack of physiological feedback in these cells. Since the original SCN2.2 cells could synchronise the fibroblast rhythms in co-culture models^52^ and the population average of the mitochondrial Ca^2+^ concentrations demonstrated circadian variations^53^, it is possible that other cell populations in SCN2.2 cells are critical for their function as a circadian pacemaker. However, the complete lack of functional sodium channels in SCN2.2YC cells and randomly selected original SCN2.2 cells (personal communication from Charles N. Allen, Oregon Health & Science University) suggests limitations to the use of these cells as a model for developed SCN neurons.

One ultimate goal of studying SCN2.2 cells is to determine the key factors that enable the recovery of system-wide rhythms in SCN-lesioned rats. Thus, the issue of whether or not implantation of SCN2.2YC cells is sufficient for the recovery of locomotor activity rhythms in SCN-lesioned rats is an important aspect for investigation, albeit beyond the scope of the present study.

## Methods

### Generation of SCN2.2YC clones

Originally stored SCN2.2 cells (from the founding immortalised cells) were cultured on laminin-coated dishes in minimum essential medium supplemented with 10% FBS (Gibco, Carlsbad, CA), glucose (3000 μg/ml), L-glutamine (292 mg/ml) and 1% penicillin/streptomycin antibiotics (Invitrogen, Carlsbad, CA) under constant temperature (37°C) and 5% CO_2_.

Because SCN2.2 cells are resistant to neomycin, the YC3.6 gene was ligated to the multiple cloning site of a zeocin-resistant vector (pcDNA3.1/zeo; Invitrogen) and transfected into SCN2.2 cells using Lipofectamine 2000 (Invitrogen). Subsequently, the cells were cultured in medium containing zeocin (400–800 μg/ml) for cell selection. Four colonies grown from single cells were picked up by a cloning ring for further subcloning using 96-well dishes. Finally, one clone showing the highest high-K^+^-induced Ca^2+^ increase was stored and used for the following experiments.

### Differentiation of SCN2.2YC cells

For differentiation of SCN2.2YC cells, the culture medium was switched from the above standard medium to serum-free Ham's F12/DMEM. Initially, we examined the addition of the following three factors (3F): 10 μg/ml insulin, 10 nM sodium selenite and 10 μg/ml transferrin. Since SCN2.2YC cells exhibited cell divisions even after culture with 3F supplemented serum-free medium, 10 ng/ml cholera toxin was supplemented in the 3F medium (4F). In addition, 10 ng/ml PMA was supplemented in the 4F medium (5F). The cell division was significantly reduced and the cell shape was polarised by the 4F medium, and the cells survived for more than 10 days after replacement of the culture medium with 5F.

### Isolated astrocyte cultures from the SCN

The experimental procedures below regarding the use of animals were approved by the Institutional Animal Care and Use Committee of the University of Toyama and Korea University. Primary cultures of isolated astrocytes were prepared from Sprague-Dawley rats at postnatal days 2–3 by the following method. Under deep anaesthesia with an intraperitoneal injection of sodium pentobarbital (50 mg/kg body weight), the brains were removed and coronal hypothalamic slices (400-μm) containing the SCN were prepared using a vibrating blade microtome in ice-cold high-Mg^2+^ artificial cerebrospinal fluid (ACSF), as described previously^12^. Subsequently, trimmed SCN tissues from eight pups were pooled in DMEM containing 100 μg/mL penicillin-streptomycin (Sigma-Aldrich, St. Louis, MO) and gently agitated in DMEM supplemented with 0.025% (w/v) trypsin (Sigma) for 15 min at 37°C in a water bath shaker. The trypsinisation was stopped by adding an equal volume of standard DMEM. A single-cell suspension of the tissue was prepared by gentle trituration and passage through a 40-μm nylon mesh cell strainer (BD Falcon, Bedford, MA), and seeded into standard culture flasks. The resultant primary cultures of SCN neurons and astrocytes were placed in a humidified CO_2_ incubator at 37°C. The medium was changed every 4–5 days until the cells reached confluency (approximately 15 days). Subsequently, the cultures were treated with a high-K^+^ buffered salt solution (BSS) consisting of (in mM) 73 NaCl, 60 KCl, 2.7 CaCl_2_, 1.2 MgCl_2_, 1 Na_2_HPO_4_, 10 glucose and 10 HEPES/NaOH (pH 7.3) for 1 h to induce neuronal cell death. Adhesive cells were passaged onto new flasks to generate SCN astrocytes. Finally, the cultures were characterised by immunostaining for GFAP.

### Real-time RT-PCR assay

Adult Sprague-Dawley rats (300–450 g; 60–70 days of age) were maintained on a 12-h/12-h light/dark cycle at a constant ambient temperature (25 ± 1°C). Food and water were available *ad libitum*. Male newborn pups underwent enucleation surgery or a sham operation under hypothermic anaesthesia on ice (10 min). A total of 40,000 U of penicillin G potassium (Sigma) was locally applied to the incisions. Finally, the pups were odorised with nesting chips and returned to their dam. For brain sampling, animals were deeply anesthetised with an intraperitoneal injection of sodium pentobarbital (50 mg/kg body weight). The whole brains were then removed and directly frozen on dry ice. The frozen brains were transferred to a cryostat chamber at −30°C, mounted with OCT compound at −15°C and sectioned at 100-μm thickness. The frozen sections were transferred onto glass slides, punched out using flat-top stainless pipettes (inner diameter, 0.33 mm; hand-made from 23 G disposable syringe tips) on ice and homogenised using a bio-masher (Funakoshi, Tokyo, Japan) in 600 μl of RLT buffer (RNeasy Kit; Qiagen, Chatsworth, CA) at 2500 rpm for 30 s. After addition of 600 μl of 70% ethanol, the samples were stored at −80°C. SCN2.2YC cells or SCN astrocytes in 25 mm^2^ flasks at 90% confluence were rinsed, suspended in PBS, transferred to 1.5 ml RNase-free tubes and centrifuged for 5 min at 1000 rpm at room temperature. The cell pellets were transferred to 350 μl of RLT buffer and homogenised using an ultrasonic cell disruptor. The resultant cell lysates were diluted with an equivalent volume of 70% ethanol and stored at −80°C until RNA extraction.

Total RNA (4 μg/sample) was extracted from tissue homogenates using the RNeasy Kit according to the manufacturer's instructions. The following PCR primers were used: *5-HT1A receptor* forward, 5′-CGTGCACCATCAGCAAGGA-3′; *5-HT1A receptor* reverse, 5′-CTGAAGATGCGCCCGTAGAGA-3′; *5-HT2A receptor* forward, 5′-ACCGCTATGTCGCCATCCA-3′; *5-HT2A receptor* reverse, 5′-GACCTTCGAATCATCCTGTAGTCCA-3′; *5-HT2B receptor* forward, 5′-CATGCATCTCTGTGCCATTTCC-3′; *5-HT2B receptor* reverse, 5′-GCGTTGACCACATCAGCCTCTA-3′; *5-HT2C receptor* forward, 5′-ACCCGTTCCCAGTAACTGTGTTTC-3′; *5-HT2C receptor* reverse, 5′-TCCCATTCCAAGGTGTGCAA-3′; *5-HT5A receptor* forward, 5′-GAAGATTTACAAGGCTGCGAAGT-3′; *5-HT5A receptor* reverse, 5′-GCGTGACGGACAGTGAACA-3′; *5-HT7 receptor* forward, 5′-CAGTACCGGAATATCAACCGGAAG-3′; *5-HT7 receptor* reverse, 5′-CCAGGGTTCCACTCTGGATCA-3′; *Per2* forward, 5′-AGCAGTCCCCTACAGCTTAACCT-3′; *Per2* reverse, 5′-CCGAGATGCGCCAGATGT-3′; *MAP2* forward, 5′-CCTCTTCTGGAAGCATCAAC-3′; *MAP2* reverse, 5′-GTAACAATTACTACAGTTGG-3′; *calretinin* forward, 5′-CTGGAGAAGGCAAGGAAAGGT-3′; *calretinin* reverse, 5′-GGCAGAGCTGGCGCAGATCC-3′; *calbindin-D28k* forward, 5′-GACGCTGACGGAAGTGGTTAC-3′; *calbindin-D28k* reverse, 5′-TCCGGTGATAGCTCCAATCC-3′; *GFAP* forward, 5′-TGGCCACCAGTAACATGCAA-3′; *GFAP* reverse, 5′-CAGTTGGCGGCGATAGTCAT-3′; *GAPDH* forward, 5′-GGCACAGTCAAGGCTGAGAATG-3′; and *GAPDH* reverse, 5′-ATGGTGGTGAAGACGCCAGTA-3′. Each primer (100 μM) was used in Rotor-Gene SYBR Green RT-PCR Master Mix (Qiagen) according to standard methods. Finally, the PCR amplification was monitored in a strip tube (25-μl reaction volume) set in the 72-well rotor of a real-time PCR system (Rotor Gene 3000A; Corbett Research, Sydney, Australia) with the following temperature steps: reverse transcription at 55°C for 10 min (Hold 1): initial PCR activation at 95°C for 5 min (Hold 2); and 60 thermal cycles of 95°C for 5 s and 60°C for 10 s. The reactions in four separate tubes were averaged for each sample.

### Electrophysiology

SCN slices (300-μm thickness) were prepared as described above using a vibrating blade microtome from male Wistar rats (12–16 days of age) and preincubated in a chamber with oxygenated normal ACSF containing (in mM) 124 NaCl, 3 KCl, 2.5 CaCl_2_, 1 MgCl_2_, 1.25 NaH_2_PO_4_, 26 NaHCO_3_ and 10 glucose for 1 h at room temperature. Subsequently, the slices were transferred to an upright microscope stage (BX-50WI; Olympus, Tokyo, Japan). The recording chamber was perfused with oxygenated normal ACSF at 1 ml/min at room temperature. SCN neurons were visualised through an infrared charge-coupled device (CCD) camera (C2741-79; Hamamatsu Photonics, Hamamatsu, Japan). The electrodes were filled with internal solution containing (in mM) 150 CsCH_3_SO_3_, 5 KCl, 5 HEPES, 0.1 EGTA, 3 Mg-ATP and 0.4 Na-GTP (pH 7.3; electrode resistances: 3–6 MΩ). Neurons were recorded in the whole-cell voltage-clamp mode with a holding potential of −60 mV using a patch-clamp amplifier (Axopatch 200B; Axon Instruments, Union City, CA). Series resistance was compensated. The output of the amplifier was digitised using an A/D converter board (Digidata 1200; Axon Instruments) with a sampling rate of 10 kHz, and recorded on a hard disk by data acquisition software (pCLAMP 8; Axon Instruments). Membrane potentials were low-pass-filtered at 2 kHz.

### Ca^2+^ imaging

The methods for *fura-2*-based Ca^2+^ imaging using acute SCN slices were described previously^12^. The methods for YC imaging using SCN slice cultures were also described previously^33^,^47^. For SCN2.2YC cell imaging, the cells were seeded onto laminin-coated glass-bottom dishes (35-mm) and cultured as described above in a CO_2_ incubator (cell density: 1–3 × 10^5^ cells/dish). The culture medium was gently rinsed from the dishes using standard BSS consisting of (in mM) 128 NaCl, 5 KCl, 2.7 CaCl_2_, 1.2 MgCl_2_, 1 Na_2_HPO_4_, 10 glucose and 10 HEPES/NaOH (pH 7.3). SCN2.2YC cells were placed on a microscope stage and continuously perfused with BSS at a flow rate of 2 ml/min through an in-line heater (SF-28; Warner Instruments, Hamden, CT) set at 36°C. 5-HT receptor agonists and antagonists were delivered to the cells by switching the perfusate.

### *Per1*-luciferase imaging

To monitor the clock gene transcriptional rhythms in SCN2.2YC cells, the cells were plated on laminin-coated 35-mm glass-bottom dishes and transfected with rat *Per1*-luciferase expression vectors (kindly donated by Dr. Hajime Tei, Kanazawa University). The culture medium was switched to medium supplemented with 1 mM beetle luciferin (Promega, Madison, WI) for 1–2 h prior to recording. The cultures were then incubated in a temperature-controlled (35 ± 0.5°C) custom-built chamber attached to an inverted microscope stage (TE-2000s; Nikon, Tokyo, Japan). The bioluminescence images were viewed using a 20× objective lens (PlanAPO VC20 × NA0.75; Nikon) and collected with a 30 min exposure time on a cooled EM-CCD camera (Cascade ll 512B; Photometrics, Tucson, AZ). Images were acquired using digital imaging software (Image Pro-Plus; Media Cybernetics, Bethesda, MD).

### Statistical analysis

Data are presented as means ± standard error. Two-way ANOVA or one-way ANOVA followed by Duncan's multiple range test was used for statistical comparisons across multiple means. A two-tailed Student's *t*-test was used for pairwise comparisons. A 95% confidence level was considered to indicate statistical significance.

## Author Contributions

K.T. and M.I. designed the study and wrote the manuscript. K.T., S.M., T.O., E.M., K.K., J.K., B.J. and J.H.H. performed the experiments. K.J.L. and M.I. directed the project and edited the manuscript.
